# Destructive effects of smoking on molecular and genetic factors of periodontal disease

**DOI:** 10.1186/1617-9625-8-4

**Published:** 2010-02-20

**Authors:** Miki Ojima, Takashi Hanioka

**Affiliations:** 1 Department of Preventive Dentistry, Graduate School of Dentistry, Osaka University, 1-8 Yamadaoka, Suita, Osaka 565-0871, Japan; 2 Department of Preventive and Public Health Dentistry, Fukuoka Dental College, 2-15-1 Tamura, Sawara-ku, Fukuoka 814-0193, Japan

## Abstract

Many epidemiological evidences have proven the association between smoking and periodontal disease. The causality can be further established by linking findings of traditional epidemiological studies with the developments in molecular techniques that occurred in the last decade. The present article reviews recent studies that address the effect of smoking on molecular and genetic factors in periodontal disease. Most findings support the fact that tobacco smoking modulates destruction of the periodontium through different pathways: microcirculatory and host immune systems, connective tissue, and bone metabolism. Although smokers experience an increased burden of inflammatory responses to microbial challenges compared to non-smokers, understanding the association between smoking and periodontal diseases involves substantial problems with respect to accuracy of measurements, and particularly, sampling of many subjects. It remains unclear whether genetic susceptibility to periodontal disease is influenced by exposure to smoking or the effect of smoking on periodontal disease is influenced by genetic susceptibility. Employment of molecular techniques may play a key role in further elucidation of mechanisms linking smoking and periodontal destruction, the direct relationship as environmental factors and indirect relationship through genetic factors.

## Background

Periodontal disease is defined as inflammatory destruction of periodontal tissue and alveolar bone supporting the teeth. Severe and prolonged periodontal inflammation leads to loss of teeth, thereby affecting oral functions (e.g., mastication, speech, and facial esthetics). Progression and severity of the disease depends on complex interactions between several risk factors such as microbial, immunological, environmental, and genetic factors, as well as age, sex, and race [1]. Tobacco smoking is a significant risk factor for periodontal disease [2].

Epidemiological studies concerning the association between smoking and periodontal disease have markedly increased since the 1990s. Based on epidemiological articles published from 1965 to 2000, the US Surgeon General's Report 2004, which comprehensively addressed active smoking and health issues, concluded that there is sufficient evidence to infer a causal relationship between smoking and periodontal disease [3]. Although biological plausibility is an important criterion in the Bradford-Hill criteria for assigning causality to an association [4], traditional epidemiology correlates exposure with disease outcomes, and everything between the cause and outcome is treated as a "black box" [5].

Despite numerous studies having demonstrated the causal association between smoking and periodontal disease, many questions remain unanswered. For example, what happens when periodontal tissue is exposed to tobacco smoke? How is the onset or progression of periodontal disease in smokers different from that in non-smokers? The underlying mechanisms of smoking-attributed periodontal disease can be further clarified by linking findings of traditional epidemiological studies with those of *in vitro *studies. Recently, molecular, cellular, and other biological markers (called biomarkers) have been frequently measured in epidemiological studies to reveal the mechanisms and events occurring along the theoretical continuum between exposure to tobacco smoke and the disease.

These biomarkers can be categorized according to the target of qualification, i.e., host responses and genetic factors (Table 1). Host responses can be further grouped as the microcirculatory system, host immune inflammatory response system, and connective tissue and bone metabolism. Since the application of a sampling technique to obtain an informative biomarker is limited, particularly in non-diseased smokers [6], saliva, blood serum, and gingival crevicular fluid (GCF) are used as specimens.

**Table 1:** Biomarkers employed in studies on smoking and periodontal disease

Target of qualification	Biomarkers	Specimens
Host responses		
Microcirculatory system	Microcirculatory functions and intercellular adhesion molecule	Gingival microvasculature
Host immune inflammatory response systems	Immune cells and immunoglobulins	Blood serum, saliva, gingival crevicular fluid, and gingival tissue
Connective tissue and bone metabolism	Cytokines, prostanoids, and matrix metalloproteinase	
Genetic factors	Genotypes associated with the immune system, inflammation, and tissue metabolism	Blood, buccal swabs, and saliva

Biological mechanisms of periodontal diseases are characterized by imbalance between bacterial virulence and host defense activity. The most plausible mechanism that explains the relationship between smoking and periodontal disease is that smoking, an environmental factor, interacts with host cells and affects inflammatory responses to microbial challenge [7]. Alternatively, the toxic components of tobacco smoke, e.g., nicotine, may directly or indirectly deteriorate periodontal tissue. Recently, genetic susceptibility to periodontal disease has been receiving much attention with respect to smoking-periodontal disease relationships. This review describes smoking as an environmental factor of periodontal disease and the interrelationship between smoking and genetic factors in periodontal disease in studies using molecular biology techniques.

## Search strategy

The PubMed database was examined for English language publications from 1965 to 2008, using the following key words: "smoking," "smokers," "tobacco," "periodontal diseases," and "periodontitis." These terms were mainly searched as title words. Epidemiological studies not employing biological measurements and review articles were excluded. Most of the articles were published in the last 10-15 years. Consequently, 134 studies were evaluated and 60 have been included in this review.

## Inflammatory host responses

### Microcirculatory System

Changes in vascular formations and microcirculatory functions in periodontal tissue following smoking can influence immune function and the subsequent inflammatory reaction in the gingiva. A significantly smaller number of vessels were observed in the inflamed gingival tissues of smokers compared to non-smokers [8]. Continuous smoking has a long-term effect that impairs the vasculature of periodontal tissue. Acute exposure to cigarette smoke induces gingival hyperemia, which is caused by the concomitant increase in blood pressure against a small but significant sympathetically induced vasoconstriction in healthy gingiva [9]. Smoking even one cigarette may cause a decrease in gingival blood flow and vascular conductance [10]. Small repeated vasoconstrictive attacks and impairment of revascularization due to cigarette smoking may contribute to disruption of the immune response and delay in the healing response, leading to an increased risk of periodontal disease. Gingival blood flow in periodontally healthy regular smokers significantly increased three days after quitting, and further small increases occurred until eight weeks compared to the baseline [11].

Vascular dysfunction may be related to impairment of oxygen delivery to gingival tissue. Smokers exhibited lower function of oxygen sufficiency in healthy gingiva and reduced ability to adapt to the function in inflamed gingiva, compared to non-smokers [12]. Pocket oxygen tension was significantly lower in smokers than in non-smokers, possibly due to impaired microcirculatory function. Correlation of pocket oxygen tension to gingival oxygen saturation of hemoglobin was highly significant in non-smokers, but this association was absent in smokers [13].

Smoking-induced endothelial dysfunction may lead to inflammatory activation within the vascular wall, mediated by cytokines and adhesion molecules. Intercellular adhesion molecule-1 (ICAM-1) is expressed on the cell surface of the endothelium of the gingival vasculature and in the junctional epithelium; it is critical in leukocyte trafficking through gingival tissue. The level of soluble ICAM-1 (sICAM-1) was higher in smokers than in age-matched non-smoking controls [14]. The mean serum sICAM-1 concentration was elevated in smokers compared to non-smokers. Conversely, the mean concentration of sICAM-1 in GCF of subjects with periodontitis was significantly lower in smokers than in non-smokers [15].

### Host Immune System

The number of neutrophils in GCF was lower or remained constant in smokers compared to non-smokers [16]. However, smoking can affect neutrophil increase in blood in a dose-dependent manner [17]. Deleterious effects of smoking on the function of polymorphonuclear neutrophils, including reduced viability and phagocytosis, were observed in periodontally healthy smokers, in a dose-response manner [18]. Although there are some conflicting data, smoking may alter neutrophil behavior in periodontal tissue. It is reported that lung macrophages are functionally compromised, e.g., reduction in capacity to produce cytokines and phagocytize microorganisms [19].

Limited evidence suggests that smoking may influence lymphocyte numbers and antibody production. In a previous study, smoking was significantly associated with an increased number of CD3+ and CD4+ T cells with a clear dose-response effect, whereas CD19+ B cells were not affected by smoking [20]. The CD4+ and CD8+ T cell values after periodontal treatment were lower in smokers than in non-smokers [21]. The serum level of IgG (Immunoglobulin G), particularly IgG2, which is an important antibody against gram-negative periodontal pathogens, was decreased in periodontitis patients who were smokers [22-27]. These findings suggest that smoking decreases the proliferative capacity of T cells or T-cell-dependent antibody responses that affect B-cell function and antibody generation.

### Connective Tissue and Bone Metabolism

Among several cytokines, levels of interleukin (IL)-1 in GCF have been extensively compared between smokers and non-smokers. Smokers exhibited significantly lower concentrations of IL-1α and IL-1ra in GCF than non-smokers [16,28]. The GCF level of IL-1β at deep bleeding sites was lower in smokers than in non-smokers [29]. This level was not different between smokers and non-smokers prior to periodontal therapy; however, it was significantly higher in smokers than in non-smokers at diseased sites following therapy [25]. Healthy smokers exhibited higher total amounts of IL-1β in GCF than non-smokers [30]. Serum IL-1β in patients with untreated aggressive periodontitis showed a positive correlation with smoking [31].

Other ILs, such as IL-4, IL-6, IL-8, and IL-10, and tumor necrosis factor-α (TNF-α) have also been investigated. The total amount of IL-4 in GCF was lower in smokers than in non-smokers and remained stable in smokers but decreased in non-smokers during induction of experimental gingivitis [32]. Smokers with early onset periodontitis exhibited lower levels of IL-4 in GCF than non-smokers [33]. The total amount of IL-10 in GCF at diseased sites was significantly lower in smokers than in non-smokers [25]; however, the levels of IL-6 and IL-8 in GCF were higher in smokers than in non-smokers [32,33]. Smokers exhibited a significantly higher level of TNF-α than non-smokers, though smoking was not associated with levels of IL-1β, IL-1ra, and IL-6 in GCF [34,35].

Considering these findings, smokers tend to exhibit excess production of inflammatory molecules, such as IL-6, IL-8, and TNF-α, and suppression of anti-inflammatory molecules, such as IL-4, IL-10, and IL-1ra; however, these findings are partly inconsistent.

IL-8 can attract and activate neutrophils. Findings regarding the effects of smoking on the level of neutrophil-derived proteolytic enzymes in oral specimens are inconsistent; however, smoking may increase their level in systemic circulation. Smokers had significantly higher elastase concentrations in GCF than non-smokers, regardless of pocket depths [36,37], while elastase concentrations decreased in smokers compared to non-smokers [38] and former smokers [18]. Plasma matrix metalloproteinase-9 (MMP-9) of smokers was 6.45 times higher than that of non-smokers [39]. Smoking was highly correlated with the MMP-3 level in GCF [40]. MMP-8 expression in periodontal tissue was significantly higher in smokers than in non-smokers [41], while the salivary MMP-8 level was significantly lower in current smokers than in former smokers [42]. Smoking may suppress the activities of protease inhibitors. Smokers had a significantly lower concentration of α-2-macroglobulin in GCF as well as total amounts of α-2-macroglobulin and α-1-antitrypsin than non-smokers [43]. Smoking seems to disturb the balance between proteolytic and anti-proteolytic activities in periodontal tissue.

IL-1, IL-6, and TNF-α stimulate the expression of the receptor activator of nuclear factor-κβ ligand (RANKL) and the inhibitor protein osteoprotegerin (OPG), which are essential factors for bone resorption and remodeling. Smoking did not affect the mean levels of free soluble RANKL (sRANKL) in GCF [44]. The OPG concentration was significantly lower and the sRANKL/OPG ratio was higher in smokers than in non-smokers, in saliva [45] as well as serum [46], explaining the greater potential for bone loss in smokers.

IL-1 and IL-6 induce production of prostaglandin E_2 _(PGE_2_) by neutrophils and macrophages, which could also promote periodontal bone resorption. However, the level of PGE_2 _in GCF in smokers was similar to that in non-smokers [47,48] or even lower than that in non-smokers [49,36]. The level of salivary PGE_2 _was also lower in smokers than in non-smokers [50]. Interference of prostaglandin production may be related to the vasoconstricting effect of smoking [51] (refer Microcirculatory system).

The level of free oxygen radicals in periodontal tissues was increased in smokers compared to non-smokers [52]. Oxidative stress induces tissue damage by injuring cells such as fibroblasts. Tobacco products inhibit attachment and growth of fibroblasts derived from human periodontal ligaments [53]. Fibroblasts impaired by smoking possibly lead to delay in tissue repair and wound healing in periodontal disease.

Smoking-associated pathophysiological changes in periodontal tissue evaluated by biological measurements are summarized in Table 2. Reduction in GCF observed in smokers may influence the conflicting results between the levels of several biomarkers in GCF and blood. It remains unclear whether these changes are due to nicotine or other components of tobacco smoke and systemic or local effects of smoking. The common mechanism in periodontal and systemic disease under the influence of smoking may be revealed by markers for inflammatory responses, tissue damage, and vascular effects [54].

**Table 2:** Pathophysiological changes associated with smoking

Target of qualification	Biomarkers*	Articles
Microcirculatory system	Gingival blood flow (chronic effect ↓ and acute effect ↑, quit effect ↑) Number of vessels in inflamed site↓ (Gingival bleeding ↓) Oxygen sufficiency ↓ Pocket oxygen tension ↓ sICAM-1 ? (Serum ↑ and GCF ↓)	9-11 8 12 13 14, 15

Host immune inflammatory response systems	PMNs or neutrophil count ? (blood↑ and GCF↓) PMN function (chemotaxis, phagocytosis, and oxidative burst) ↓ Macrophage function ↓ T lymphocytes ? IgG2 ↓	16, 17 18 19** 17, 20, 21 22-27

Connective tissue and bone metabolism	IL-1a/β and IL-1ra ↓ IL-6 and IL-8 ↑, IL-4, and IL-10 ↓,TNF-α ↑ Elastase activity ? MMP-9 and MMP-3 in GCF ↑ MMP-8 ? (GCF ↑ and saliva ↓) α-2-Macroglobulin ↓ OPG ↓ and sRANKL/OPG ratio ↑ Prostaglandin E_2 _↓ Free radicals ↑ Gingival fibroblast ↓ (Tissue repair and wound healing ↓)	16, 25, 28-31 25, 32-35 18, 36-38 39, 40 41, 42 43 44-46 36, 47-50 52 53

*↑: increase, ↓: decrease, ?: uncertain. **Lung macrophages

ICAM-1: intercellular adhesion molecule-1, GCF: gingival crevicular fluid, PMN: polymorphonuclear neutrophil, IgG: immunoglobulin G, IL: interleukin, TNF-α: tumor necrosis factor-α, MMP: matrix metalloproteinase, OPG: osteoprotegerin, RANKL: receptor activator of nuclear factor-κβ ligand

## Genetic factors

Gene polymorphisms have been investigated as possible markers of increased susceptibility to periodontal diseases: IL-1, IL-4, IL-10; TNF-α; Fcγ receptor; human leukocyte antigen; vitamin D receptor; and N-formyl peptide receptor [55]. Relationships between smoking and genetic susceptibility to periodontal diseases have been strengthened with respect to genotypes associated with cytokines (IL-1, IL-6, and IL-10), the immune system (Fcγ receptor), bone metabolism (vitamin D receptor), and xenobiotics metabolism (N-acetyltransferase and myeloperoxidase). These studies have been listed in Table 3.

**Table 3:** Target genotypes and study population in association with periodontal disease and smoking

Genotypes	Subjects	Main findings	Articles
IL-1A -889, IL-1B +3954 (originally described as +3953)	134 subjects, USA	The polymorphic IL-1 gene cluster was associated with severity of periodontitis only in non-smokers.	59
IL-1A -889, IL-1B +3954 (originally described as +3953)	28 African-American and 7 Caucasian-American families (early onset periodontitis affected and unaffected subjects), USA	IL-1ß disequilibrium with EOP was found both in smokers and non-smokers.	57
IL-1A -889	46 patients and 12 controls, UK	The carriage of allele 2 was associated with an increase in IL-l α protein levels, especially in non-smokers, while heavy smokers showed reduced levels of IL-lα protein, regardless of genotype.	28
IL-1A -4845, IL-1B -3954	295 Caucasians, Australia	A relationship was observed between the IL-1-positive genotype and increased mean probing pocket depth in non-smokers more than 50 years of age. IL-1 genotype-positive smokers had an increase in the number of probing depths ≥3.5 mm.	56
IL-1A +4845, IL-B +3954	90 patients (non- or former smokers), USA	IL-1 genotype-positive non-smokers or former light smokers were at increased odds of having moderate-to-severe periodontal disease compared to IL-1 genotype-negative patients. The presence of both former moderate smoking history and IL-1-positive genotype showed a lower likelihood of developing the disease when compared to those with presence of only one of the risk factors.	60
IL-1A -889, IL-1B +3954, IL-1RN	154 Caucasians, Germany	Severity of periodontitis was associated with the composite genotype of IL-1α/1β in smokers, while no differences were found in genotype-negative subjects, irrespective of their smoking status.	62
IL-1A -889, IL-1B +3954, IL-1B -511	1085 Caucasians, Germany	An increased risk of periodontal disease and tooth loss was observed for IL-1 genotype-positive smokers.	61, 63, 64
IL-1A -889 IL-1B +3954	330 patients and 101 controls, Chile	The association between positive genotype and periodontitis was independent of smoking status.	58
IL-6 -174	155 patients and 54 controls, Brazil	An association between the G-genotype and periodontal status was observed only in non-smokers.	65
IL-10 -1087	60 patients and 39 controls, Sweden	An association between the GG genotype and periodontal status was more conspicuous in non-smokers.	66
Vitamin D receptor -1056 Taq-I	303 patients and 231 controls, UK	Vitamin D receptor Taq-I TT polymorphism was moderately associated with both the presence and progression of periodontitis in smokers.	70
FcγRIIIb	164 subjects aged 70 years old, Japan	An association between smoking and periodontal disease progression in elderly people with FcγRIIIb-NA2 polymorphism.	68
FcγRIIa	422 Caucasians, USA	FcγRIIa-H/H131 genotype may be associated with chronic periodontitis risk in smokers.	69
FcγRIIIa -158V/F, FcγRIIIb -NA1/NA2	1083 Caucasians, Germany	Smokers show a significantly increased attachment loss in the presence of FcγRIIIb-NA2 allele. The different genotypes show no differences in non-smokers.	63
IFNGR1	62 patients and 56 controls, Norway	In combination with smoking, IFNGR1 was significantly associated with periodontitis.	67
NAT2 -T341C, -G590A, G857A MPO G-463ª	1083 Caucasians, Germany	Smokers with the high activity variant of NAT 2 and MPO are at an increased risk of periodontitis.	63

IL: interleukin, FcγR: Fcγ receptor, IFNGR1: interferon gamma receptor 1, NAT: N-acetyltransferase, MPO: myeloperoxidase

IL-1 polymorphisms have been intensively studied using a cross-sectional approach, except for one study that employed a longitudinal design [56]. The relationship with respect to smoking is controversial. The association between positive genotypes and the severity of periodontal disease was independent of smoking [57,58], suggesting no relationship between smoking and IL-1 genotypes; however, relationships between IL-1-positive genotypes and smoking was evident [59-63]. Logistic regression analysis of periodontal disease with genotype-negative non-smokers as a reference group exhibited odds ratios of 0.98 for genotype-positive non-smokers, 2.37 for genotype-negative smokers, and 4.50 for genotype-positive smokers, thus suggesting synergism between IL-1 polymorphism and smoking [64].

Non-smokers with moderate periodontitis and periodontally healthy subjects displayed a higher incidence of IL-6 G-genotype than severe periodontitis subjects [65]. The difference in the occurrence of the IL-10 GG genotype between severe chronic periodontitis and control groups was more evident in non-smokers [66]. Gene coding for the ligand-binding chain of interferon gamma receptor 1, a cytokine that plays a pivotal role in defense against infection, was significantly associated with periodontitis in combination with smoking [67]. IgG-binding factors, namely Fcγ receptors, could influence the ability of phagocytosis. Genotypes of Fcγ receptor, FcγRIIa, and FcγRIIIb, may be associated with periodontal disease in smokers [68,69]. Vitamin D receptor Taq-I TT polymorphism was moderately associated with both the presence and progression of periodontitis in smokers [70]. Gene polymorphisms for enzymes that can metabolize smoking-derived substances may contribute to individual susceptibility to the risk of periodontitis among smokers. Subjects with the polymorphic cytochrome P450 1A1 M2 allele and glutathione S-transferase M1 allele exhibited an increased risk of periodontitis [71].

## Conclusions

The process of periodontal disease is such that a microbial challenge induces a host response, which may result in connective tissue and alveolar bone destruction. Most findings support that smoking modulates the destruction of the periodontium through different pathways: microcirculatory and host immune systems, connective tissue, and bone metabolism. Although smokers exhibit an increased burden of inflammatory responses to microbial challenges compared to non-smokers, substantial problems still persists with respect to the accuracy of measurements, and particularly, the sampling of many subjects. Limited evidences are available regarding the effects of quitting smoking on pathophysiological changes in periodontal tissue.

At present, gene-smoking relationships in periodontal disease are suggestive. The reported gene-smoking relationships in periodontal disease should be carefully interpreted in two ways. First is the uncertainty of the relationship because of methodological limitations such as employment of subjects in a specific race, small sample size, and lack of detailed history of smoking and possible confounders. Further studies with adequate statistical power and small biases would be required to clarify the relationship. Furthermore, it remains unclear whether genetic susceptibility to periodontal disease is influenced by exposure to smoking or the effect of smoking on periodontal disease is influenced by genetic susceptibility. The second issue is an ethical problem. If a smoker with positive genotypes associated with inflammatory cytokines is proven to be at a high risk of periodontal disease, the evidence is so strong that the smoker should be encouraged to quit smoking. In contrast, however, the evidence may cause a smoker with negative genotypes to avoid quitting smoking, thereby causing fatal diseases. Within such limitations, gene-environment relationship studies possibly provide valuable insights into the pathogenesis of complex periodontal diseases and are expected to contribute to prevent the disease through personalized recommendation and targeted intervention in dental public and clinical programs.

Employment of molecular techniques may play a key role in further elucidation of mechanisms linking smoking and periodontal destruction, the direct relationship as environmental factors and indirect relationship through genetic factors.

## Competing interests

The authors declare that they have no competing interests.

## Authors' contributions

MO evaluated the literature and drafted the manuscript. TH conceived the idea for the review and helped draft the manuscript. Both authors participated in the design and coordination of the review. They also read and approved the final version of the manuscript.
